# Combined Reconstruction of the Anterior Cruciate Ligament and Anterolateral Ligament: Triple-Strand Braided Hamstring Graft for the Anterior Cruciate Ligament and Gracilis Strand for the Anterolateral Ligament With a Single Femoral Tunnel

**DOI:** 10.1016/j.eats.2024.103023

**Published:** 2024-05-14

**Authors:** Diego Ariel de Lima, Camilo Partezani Helito, Sergio Marinho de Gusmão Canuto

**Affiliations:** a Universidade Federal Rural do Semi-Árido (UFERSA), Mossoró, Brazil; b Hospital Tarcísio Maia (HRTM), Mossoró, Brazil; c Universidade de São Paulo, Grupo de Joelho, Instituto de Ortopedia e Traumatologia, Hospital das Clínicas, Faculdade de Medicina da Universidade de São Paulo (HCFMUSP), São Paulo, Brazil; d Hospital Sírio Libanês, São Paulo, Brazil; e Ortoclínica Hospital de Ortopedia, Maceió, Brazil; f Santa Casa de Misericórdia (SCMM), Maceió, Brazil

## Abstract

This article presents a surgical technique for combined reconstruction of the anterior cruciate ligament (ACL) and anterolateral ligament (ALL) of the knee using hamstring tendon graft. This approach involves creating a single femoral tunnel and using a triple-strand braided hamstring graft for the ACL and a strand of the gracilis tendon for the ALL. This technique aims to increase strength and improve knee stability, potentially reducing the risk of reinjury. The described method provides anatomic reconstruction of the ACL and ALL and allows for combined reconstruction with just 1 graft donor site. Another advantage is that the triple braided graft takes on a tape-like shape, mimicking the native form of the ACL and theoretically increasing biomechanical strength. Combined reconstruction of the ALL and ACL has shown excellent results in specific patient groups, reducing graft failure and improving outcomes in patients with a high risk of rupture. Thus, we describe a promising option for ligament reconstruction in cases of combined ACL and ALL injuries. This method may be a valuable alternative for orthopaedic surgeons, combining anatomic reconstruction with the use of a single donor site.

Combined reconstruction of the anterolateral ligament (ALL) and anterior cruciate ligament (ACL) of the knee has shown excellent results in specific patient groups.^1^, ^2^, ^3^, ^4^ This could potentially reduce graft failure and improve outcomes in high-risk patients. Hamstring graft is commonly used for this type of reconstruction.^5^, ^6^, ^7^, ^8^

Studies on isolated intra-articular ACL reconstruction have shown that hamstring grafts with a diameter smaller than 8 mm may have a higher risk of failure, but this is not as well established when extra-articular reconstruction is performed. Grafts of 7 mm or less, when associated with ALL reconstruction, may yield similar results to isolated intra-articular grafts of 8 mm or more.^9^ Ideally, a technique should be developed that allows for reconstruction of the ALL and provides a graft thick enough for ACL reconstruction without the need for other donor sites.

Thus, the aim of this work is to describe a technique using hamstring tendon graft for combined ACL and ALL reconstruction with a single femoral tunnel (Fig 1, Fig 2, Fig 3). This technique uses a triple-strand braid (1 strand of the gracilis tendon [GT] and 2 strands of the semitendinosus tendon [ST]) for the ACL reconstruction and leaves a “free” GT strand for the ALL reconstruction.

**Fig 1:**
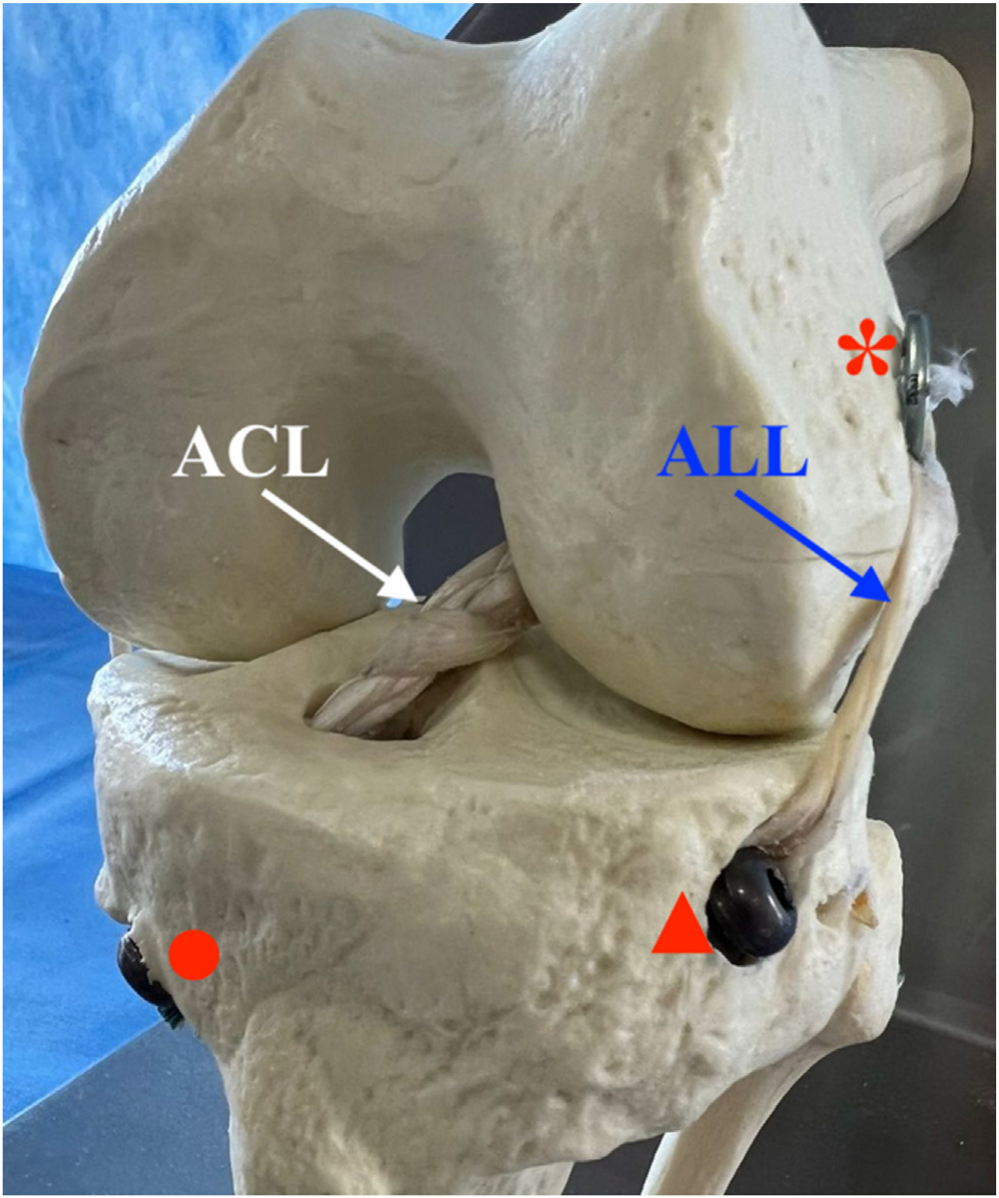
Model showing technique for anterior cruciate ligament (ACL) and anterolateral ligament (ALL) reconstruction using triple-strand braided hamstring graft for ACL and strand for ALL reconstruction. The asterisk indicates an EndoButton fixing the triple-strand braided graft in the femoral tunnel; triangle, an interference screw fixing the strand for the ALL reconstruction at its tibial insertion, between the Gerdy tubercle and the head of the fibula; and circle, an interference screw fixing the triple-strand braided graft in the tibial tunnel.

**Fig 2:**
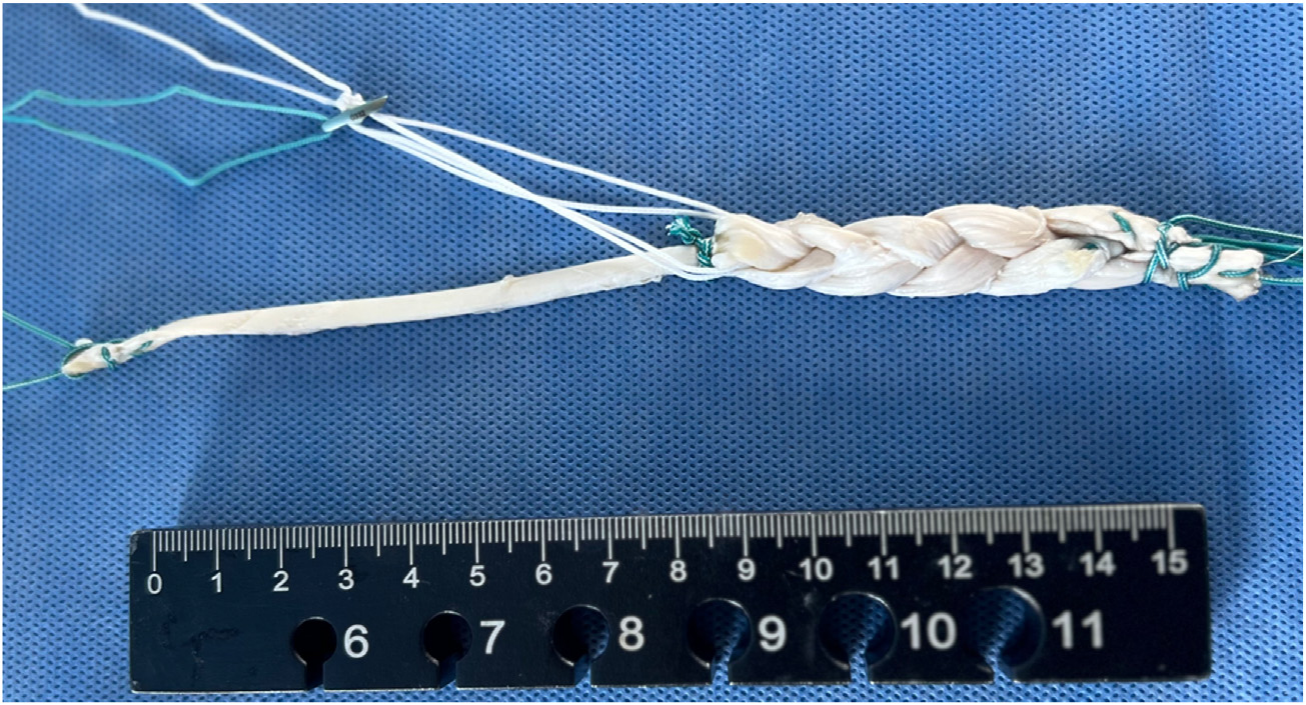
Model showing technique for triple-strand braided graft for reconstruction of anterior cruciate ligament and strand for reconstruction of anterolateral ligament. The graft is positioned in a cortical suspension fixation device such as an EndoButton plate.

**Fig 3:**
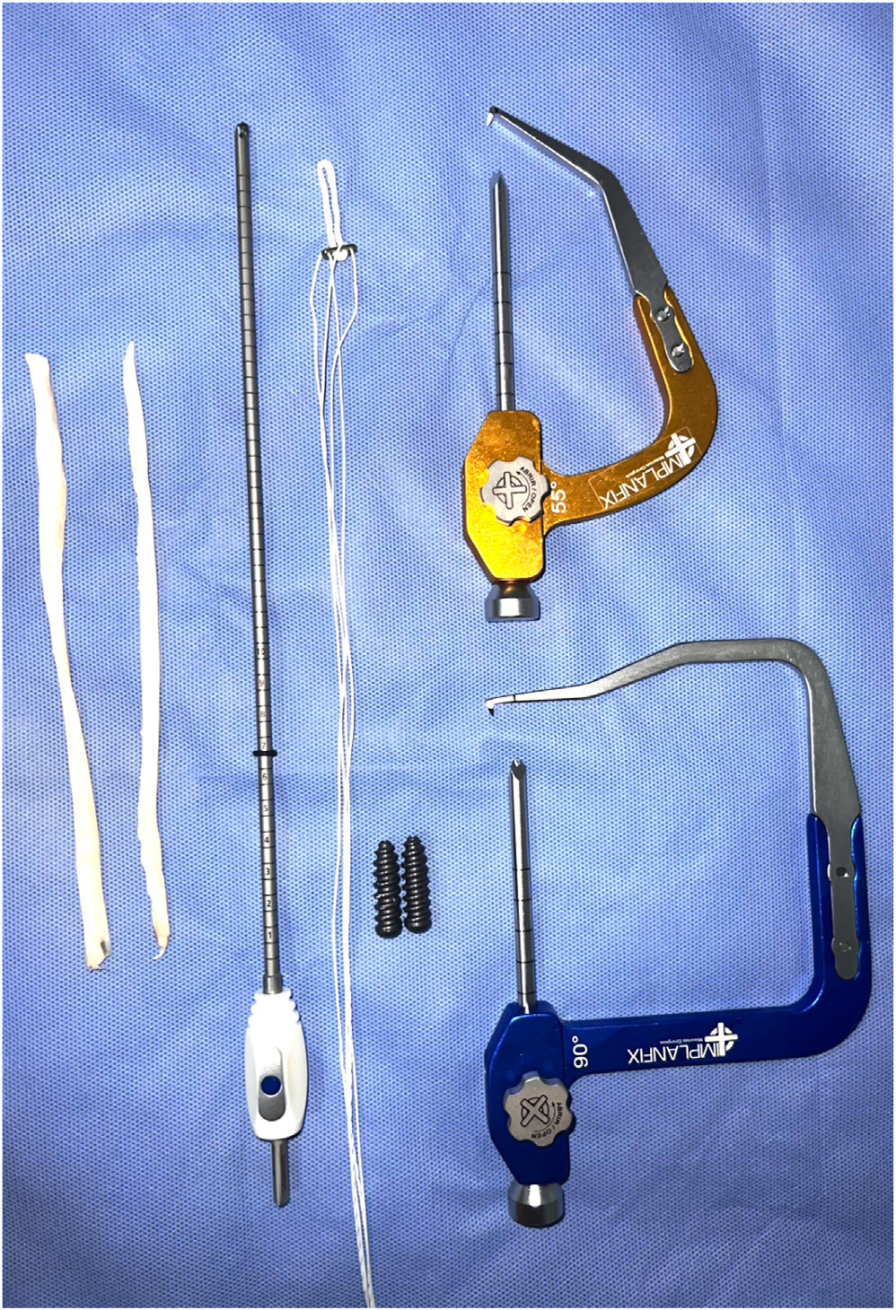
Necessary materials for procedure: 1 cortical suspension fixation device such as EndoButton plate (our preference is an adjustable-loop button model); 2 interference screws; 1 retrograde drill; tibial guide set to 55°; and femoral guide set to 90°.

## Technique

The complete technique is demonstrated in Video 1, pearls and pitfalls are presented in Table 1, and advantages and disadvantages are listed in Table 2.

**Table 1:** Pearls and Pitfalls

Pearls
Accurate indications for combined ACL and ALL reconstruction are crucial.
Imaging examination should be performed for surgical planning, especially in revision cases.
Attention should be paid to the positioning of the tendons on the button: The center of the ST should be in the loop, while the GT is slightly pulled so that 1 of its strands is the same length as the other 2 strands of the ST.
Suturing is performed to transversely fix the 3 flexor tendon strands (1 GT and 2 ST) 1 cm distal to the button loop.
During the making of the braid, it is crucial to keep the graft under tension.
Before using the retrograde drill, the surgeon should use the 4.5-mm cannulated reamer for drilling of the femoral and tibial tunnels over the guide pins.
Identification of the lateral femoral epicondyle, Gerdy tubercle, and head of the fibula is fundamental (when in doubt, fluoroscopy should be used).
Pitfalls
The creation of the tunnels is crucial, especially the femoral one. Mispositioning should be avoided.
Care should be taken when deactivating the reverse reaming mechanism in the femur: If the entire length of the femoral tunnel is enlarged with the reverse drill, this will preclude the use of the button.
The cortical suspension device should be placed before the braid configuration is started.
Inadequate continuous tension and angles during the preparation of the braid can lead to a nonuniform final graft configuration.
A minimum length of 8-9 cm is desirable for secure fixation of the final graft.
Care should be taken when cutting the sutures of the suspension device (button) after femoral and tibial fixation to prevent the device from turning.

ACL, anterior cruciate ligament; ALL, anterolateral ligament; GT, gracilis tendon; ST, semitendinosus tendon.

**Table 2:** Advantages and Disadvantages

Advantages
Anatomic reconstruction of the ACL and ALL is provided.
Combined reconstruction is achieved with just 1 graft donor site.
Triple braided graft takes on a tape-like shape, mimicking the native form of the ACL and theoretically increasing biomechanical strength.
The technique can be used with fixed- or adjustable-loop cortical suspension devices.
Disadvantages
The surgical procedure is more complicated and expensive.
The braid causes a shortening of approximately 5-10 mm in the length of the triple graft.
The technique requires at least 2 operators to perform braiding on an auxiliary table to maintain appropriate tension.
Because the braid is made manually, there is no standardization of how tight the braid is.

ACL, anterior cruciate ligament; ALL, anterolateral ligament.

### Surgical Indications

The main surgical indications described for combined ACL reconstruction and ALL reconstruction are ACL revision surgery, physical examination with grade 2 or 3 pivot shift, participation in sports involving pivoting mechanisms and/or high-level activity, ligamentous laxity, and Segond fracture; secondary indications may include chronic ACL injury, age under 25 years, and radiologic signs of lateral femoral condyle depression.^3^

### Necessary Materials for Procedure

The materials needed for the procedure are as follows: 1 cortical suspension fixation device such as an EndoButton plate (Smith & Nephew); 2 interference screws; 1 retrograde drill, such as the FlipCutter (Arthrex), Tunneling Drill (Razek), Infinity Knee System (ConMed), or Acufex Trunav Retrograde Drill (Smith & Nephew); 1 cannulated reamer/drill (4.5 mm); a 90° femoral guide (such as the Chambat guide); a 55° tibial guide; 2 guide pins (2 mm); a tendon stripper; and basic materials for arthroscopy (Fig 3). Regarding the cortical suspension fixation device, our preference is an adjustable-loop button model, such as the ACL TightRope (Arthrex), FastFit Button (Razek), GraftMax (ConMed), or Ultrabutton (Smith & Nephew).

### Graft Harvest

An incision of approximately 2 to 3 cm is made over the insertion of the pes anserinus tendons, medially to the tibial tubercle. Subsequently, an oblique incision in the sartorius fascia is made to expose the hamstring tendons. The GT and ST are carefully dissected and released distally at the tibial tubercle. The tendons are then sutured at the ends with Ethibond Excel thread (Ethicon) to facilitate subsequent handling. The GT and ST are dissected and released from fascial adhesions. A tendon stripper is used to release each tendon from its proximal muscular attachment while flexing the knee in a slight varus motion.

### Preparation of Triple-Strand Braided Graft

**Fig 4:**
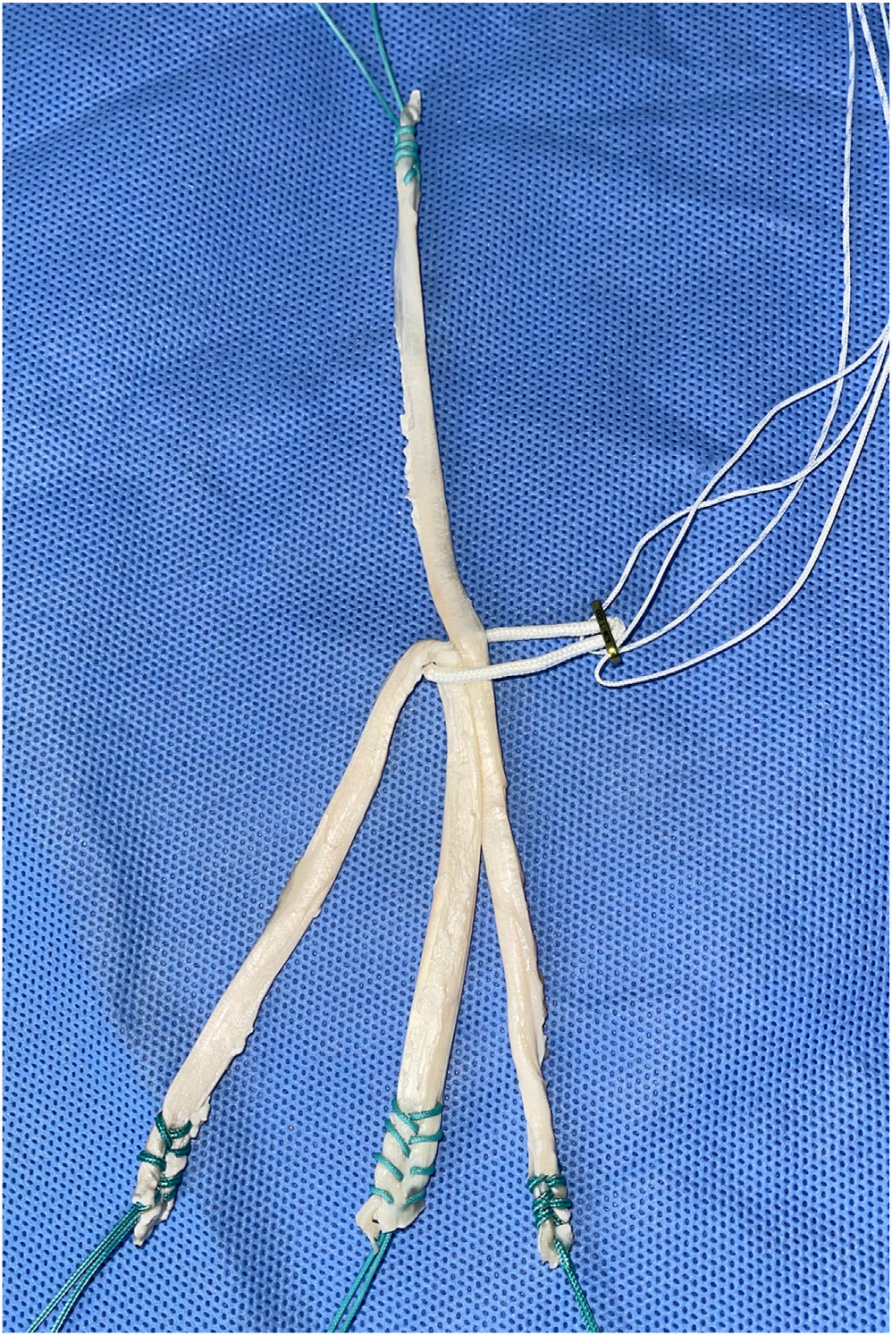
Triple-strand hamstring graft before braiding. Model showing technique for triple-strand braided graft for reconstruction of anterior cruciate ligament and strand for reconstruction of anterolateral ligament. The graft is positioned in a cortical suspension fixation device such as an EndoButton plate.

The autograft obtained is placed on the preparation table, where excess muscle tissue and worn parts of the tendons are removed. The other ends of the tendons are then also sutured with Ethibond thread. Each tendon is folded in half to form 4 strands. After the tendons are folded at the midpoint, they are positioned in a cortical suspension fixation device such as an EndoButton plate. Our preference is an adjustable-loop button model. At this point, one of the main steps of graft preparation is performed. The GT is unfolded and gently pulled so that 1 of its strands is the same length as the 2 other strands of the ST (to produce a uniform braid with all 3 strands of similar length). Thus, we end up with 1 free GT strand and 3 strands (1 GT and 2 ST) positioned on the button (Fig 4).

A No. 0 Vicryl suture (Ethicon) is used to transversely fix the 3 flexor tendon strands (1 GT and 2 ST) 1 cm distal to the button loop, preventing slippage during the braiding process. The proximal end of each strand is kept stretched during the making of the braid.

**Fig 5:**
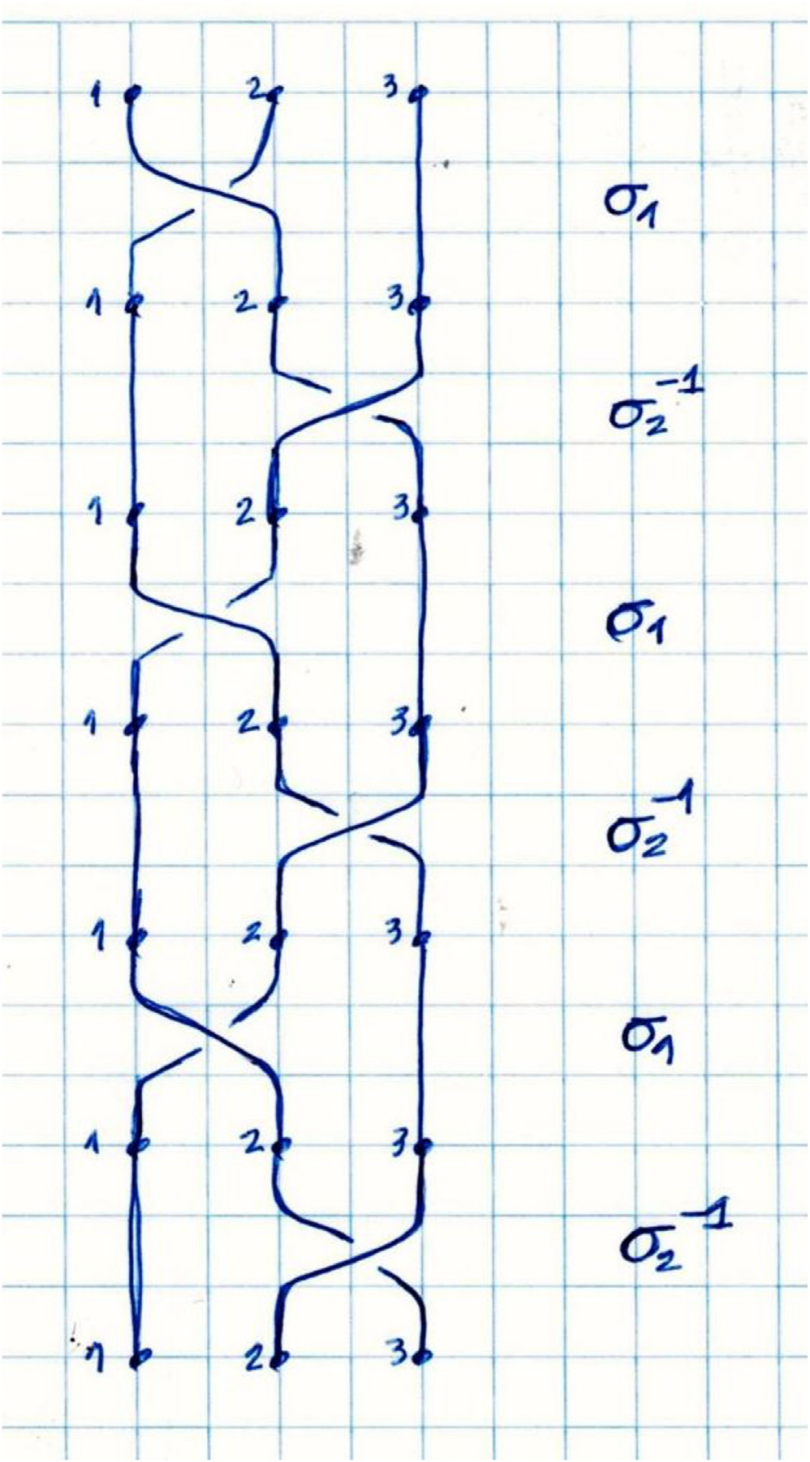
Pure-form braid.^10^ Model showing technique for triple-strand braided. σ__1__, exchange between strands 1 and 2; σ__2__, exchange between strands 2 and 3.

**Fig 6:**
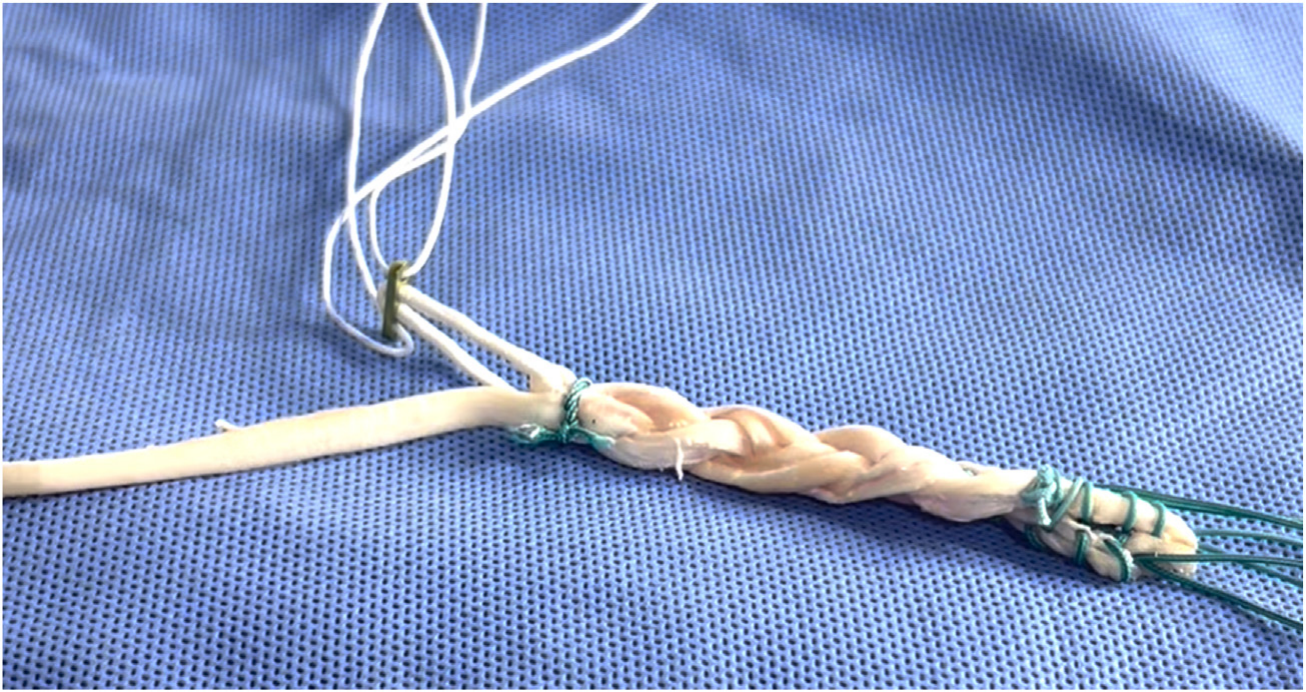
Triple-strand braided hamstring graft after braiding. Model showing technique for triple-strand braided graft for reconstruction of anterior cruciate ligament and strand for reconstruction of anterolateral ligament. The graft is positioned in a cortical suspension fixation device such as an EndoButton plate.

Measurements of length and diameter are taken before configuring the triple braid of the hamstring. The triply united graft (1 GT and 2 ST) is braided in a “pure” form: (σ_1_σ_2_^−1^)3*n*, with *n* being a positive integer, that is, the sequence of concatenations σ_1_σ_2_^−1^ σ_1_σ_2_^−1^ σ_1_σ_2_^−1^ repeated an integer number of times (Figs 5 and 6).^10^ These steps are repeated until the end of the graft, and the strands are then sutured together. It is crucial to maintain the braided hamstring graft under tension. The final dimensions of the graft are measured in terms of length and diameter. After graft preparation, the arthroscopic part of the procedure begins through the anteromedial and anterolateral portals.

### Tibial Tunnel

The center of the tibial tunnel should be at its native insertion, approximately aligned with the posterior edge of the anterior horn of the lateral meniscus, about 15 to 20 mm anterior to the posterior cruciate ligament.^11^ We use a tibial guide set to 55° and positioned with its intra-articular aiming tip through the anteromedial portal, at the center of the ACL footprint, as described earlier. Extra-articularly, through the same access used for hamstring graft harvest, the guide is adjusted so that the center of the tunnel is approximately 4 cm from the tibial joint line (about 1 cm above the hamstring insertion on the pes anserinus) and 2 cm medial to the tibial tuberosity. A 2-mm guide pin is then advanced through the guide until the tip is visible intra-articularly.

### Femoral Tunnel

The center of the femoral tunnel is at its native insertion, in the lateral femoral condyle, posterior to the resident’s ridge.^11^ We use a 90° femoral guide, positioned with its intra-articular aiming tip through the anterolateral portal, at the center of the ACL footprint, as described earlier. Extra-articularly, through a longitudinal access of approximately 2 cm at the topography of the lateral femoral epicondyle, the guide is adjusted so that the center of the tunnel is near the femoral origin of the ALL, posterior and proximal to the lateral femoral epicondyle by about 4 mm and 8 mm, respectively.^12^, ^13^, ^14^ A 2-mm guide pin is then advanced through the guide until the tip is visible intra-articularly.

With the knee at approximately 90° of flexion, a 4.5-mm cannulated reamer/drill is used for drilling the femoral and tibial tunnels over the guide pins. This diameter is sufficient for the passage and “flipping” of the EndoButton plate, according to the chosen button mark.

**Fig 7:**
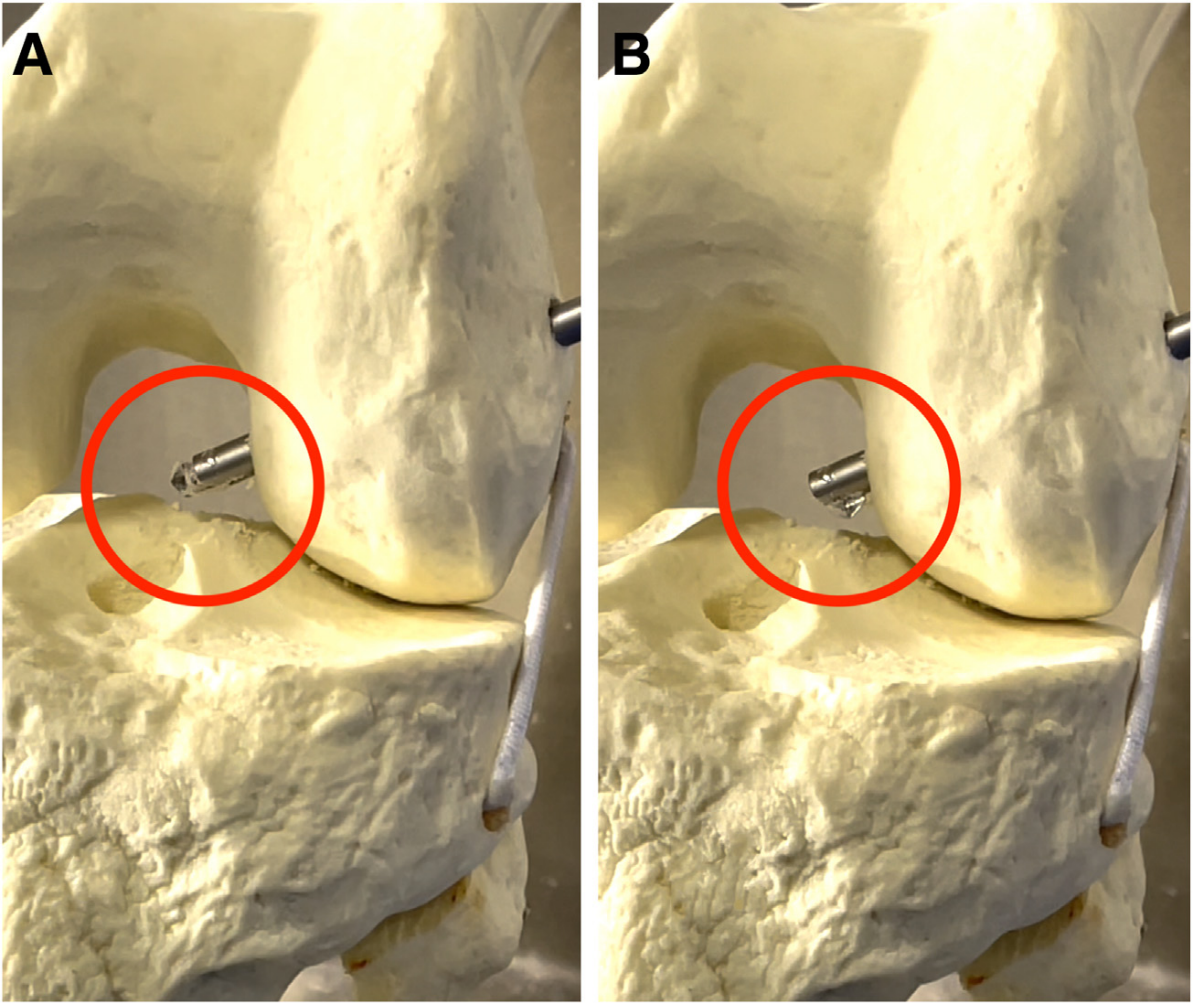
Preparation of single femoral tunnel for combined anterior cruciate ligament and anterolateral ligament reconstruction. (A) Retrograde drill (circle). (B) Reverse reaming mechanism activated (circle).

We choose the diameter of the retrograde drill depending on the diameter of the triple-strand braided graft.^15^ We start with the femoral tunnel. We pass the drill from outside to inside, through the 4.5-mm femoral tunnel created in the previous step. With the tip of the drill visible intra-articularly, we activate the reverse reaming mechanism and enlarge the tunnel from inside to outside, with a diameter equal to the braided graft and a length of 2 to 3 cm, sufficient for good healing and integration of the graft in the femoral condyle (Fig 7). We return the drill into the knee and, under direct arthroscopic vision, deactivate the reverse reaming mechanism and remove the drill from the knee.

In the tibial tunnel, the drill is passed from outside to inside, through the 4.5-mm tibial tunnel created in the previous step. With the tip of the drill visible intra-articularly, we activate the reverse reaming mechanism and enlarge the tunnel from inside to outside, with a diameter equal to the braided graft and through the entire length of the tibial tunnel, until the drill completely exits the knee. (In this step, we do not deactivate the reverse reaming mechanism, making the tibial tunnel with a diameter equal to the braided graft.)

### Passage and Fixation of Triple-Strand Braided Graft

**Fig 8:**
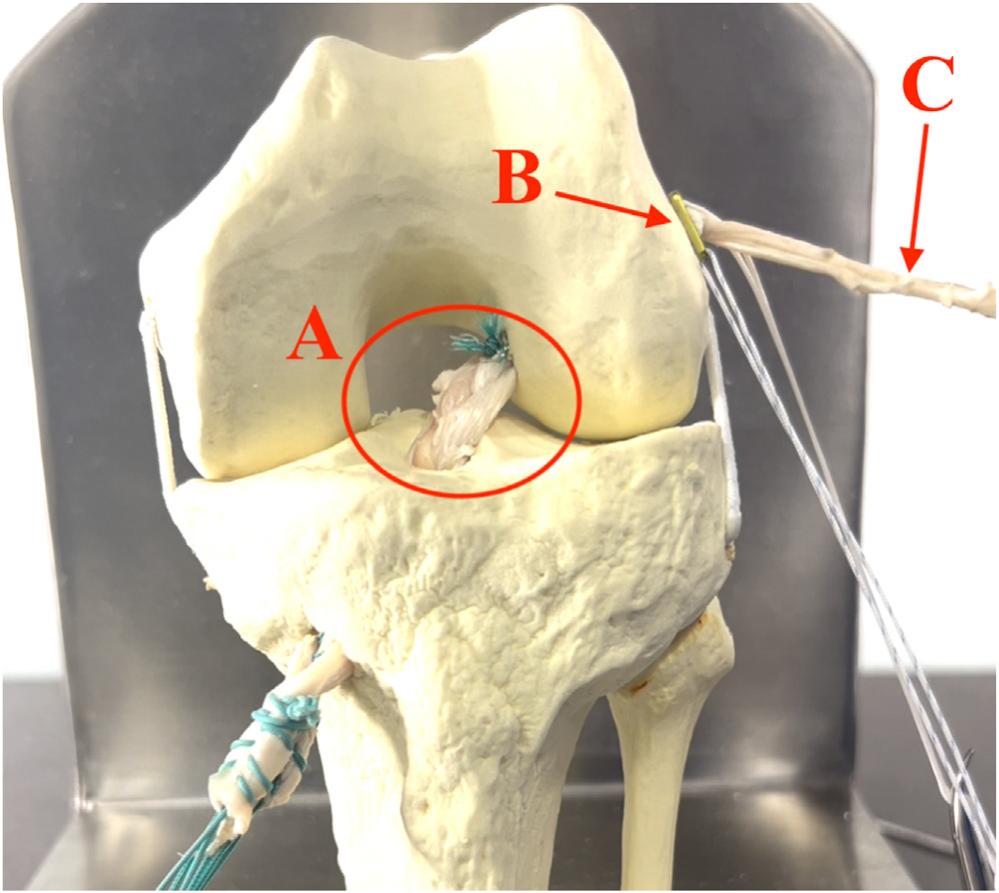
Passage and fixation of triple-strand braided graft in tibial and femoral tunnels. (A) Triple-strand braided hamstring graft for anterior cruciate ligament. (B) Button anchored against lateral femoral cortex. (C) Gracilis strand for anterolateral ligament. circle represents triple-strand braided graft.

With the help of a surgical marking pen or Vicryl suture thread, we make a mark on the triple graft at the end closest to the button, measuring 2 to 3 cm in length, as a reference. This measurement corresponds exactly to the length of the femoral tunnel made with the retrograde drill. A suture-capturing instrument (or arthroscopic grasper) is used to transport the suture from the femoral tunnel to the outside of the tibial tunnel; then, the triple braided graft, the button, and the free GT strand (for the ALL reconstruction) are passed with the suture. Once the button is firmly anchored against the lateral femoral cortex, the graft is introduced into the femoral tunnel and through the button’s adjustment system (Fig 8). At this stage, we leave our mark made with the pen (or suture thread) clearly visible intra-articularly, meaning the graft does not fill the entire femoral tunnel. (This step is important because we can still tension the graft through the button’s adjustment system after tibial fixation of the graft.) The knee is moved about 10 times to eliminate slack in the graft. Tibial fixation is secured with an interference screw, and we again use the button’s adjustment system for final positioning and tensioning of the graft.

### ALL Reconstruction

**Fig 9:**
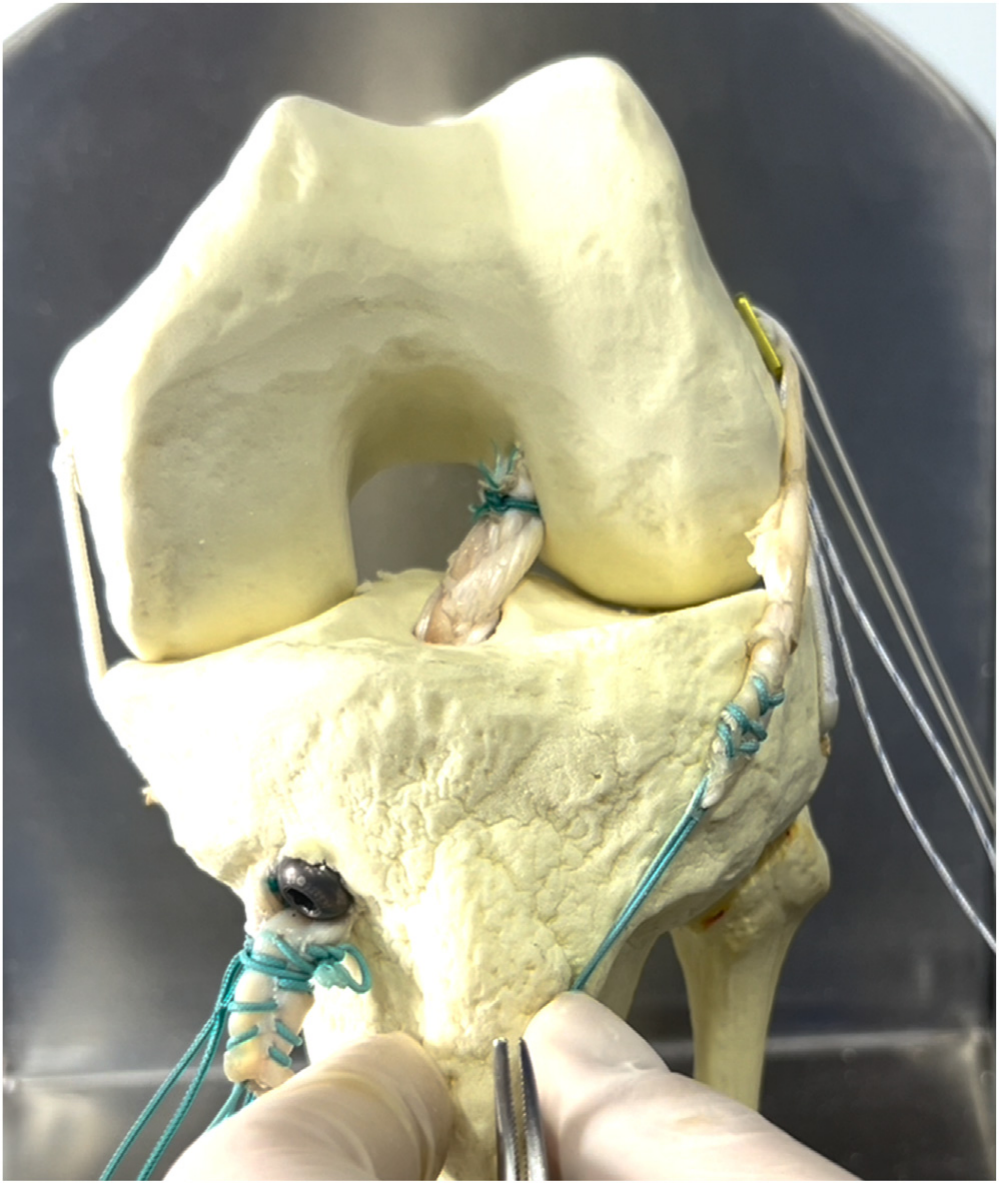
After fixation of the anterior cruciate ligament graft (triple-strand braided), the free gracilis strand is used for reconstruction of the anterolateral ligament.

After fixation of the ACL graft, the free GT strand is used for the reconstruction of the ALL (Fig 9). Subsequently, an approximately 2-cm access is made at the topography of the tibial insertion of the ALL.^12^, ^13^, ^14^ This point can be located using fluoroscopy or anatomic reference points. Our current preference is to identify this point anatomically between the head of the fibula and the Gerdy tubercle, approximately 5 to 10 mm below the articular line of the lateral tibial plateau. By use of radiographic reference points, this point is located about 7 mm below the tibial plateau in the frontal view and around 50% of the length of the plateau in the lateral view.^12^, ^13^, ^14^ The remaining portion of the gracilis then passes under the iliotibial tract to its tibial insertion point.

A 2-mm guide pin is advanced through the tibial insertion of the ALL until the tip is visible near the medial access for graft harvest. A 4.5-mm cannulated reamer is used to drill a tunnel over the guide pins. After creating this tibial tunnel, we enlarge the tunnel with a 7-mm drill at the tibial origin of the ALL, inserting only 20 to 25 mm. This measurement is a result of our smallest interference screw being 7 mm in diameter and 20 mm in length.

**Fig 10:**
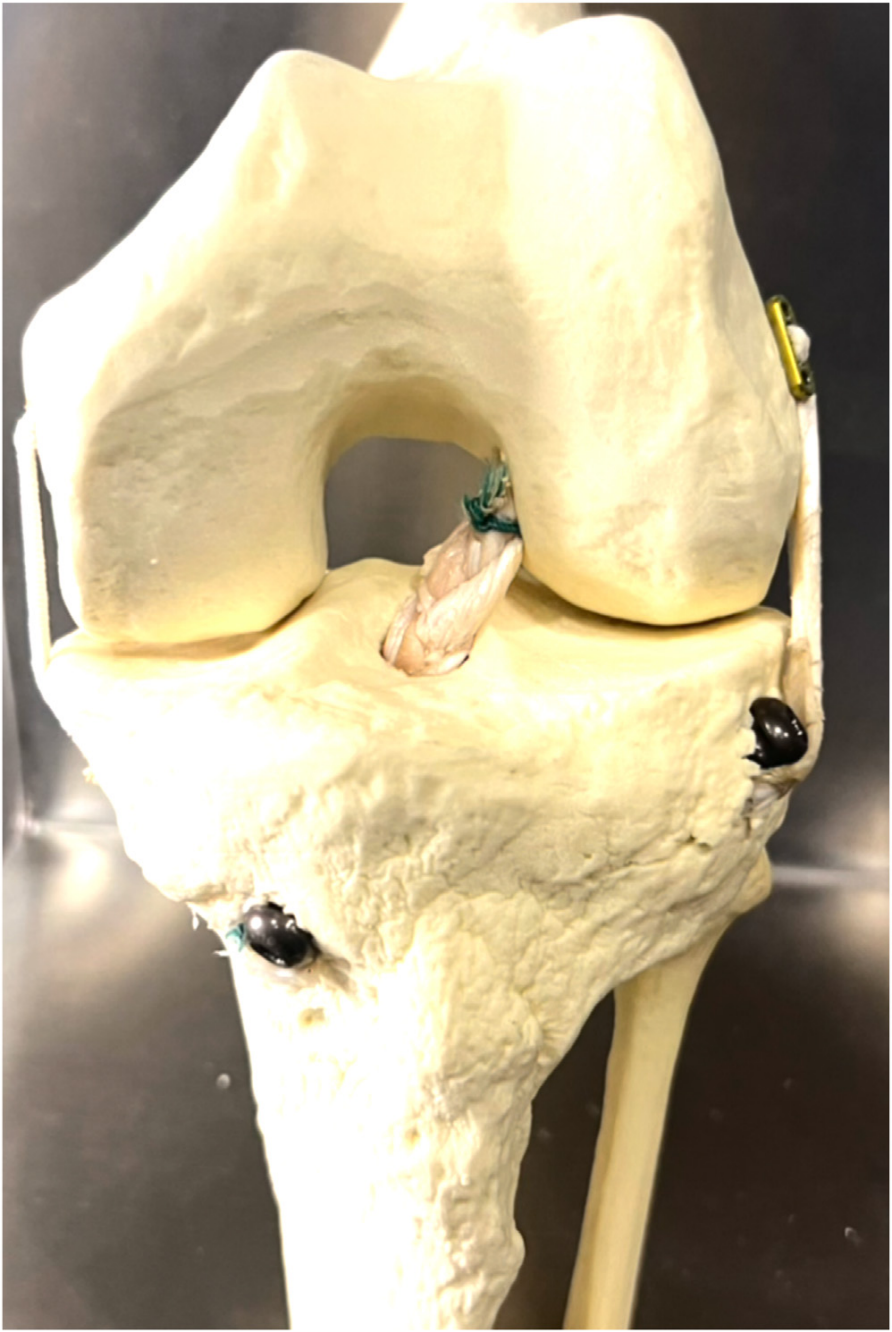
After fixation of the anterior cruciate ligament graft (triple-strand braided), fixation of the anterolateral ligament graft is performed with an interference screw.

A suture-capturing instrument is used to transport the free GT strand into the newly created tibial tunnel. Fixation of the ALL graft is performed with a 7 × 20-mm interference screw (or smaller if available) (Fig 10).

After reconstruction, closure is performed. Intraoperative fluoroscopy is used to confirm the placement of the button at the appropriate site, as well as the identification of the tunnel positioning. We do not use a suction drain or immobilization brace after reconstruction. The patient begins formal physiotherapy after the first postoperative consultation (approximately in a week).

## Discussion

The presented technique offers the main advantage of reliably reproducing the anatomy of both the ACL and ALL with hamstring graft. In the technique in question, the triple braid of hamstring graft has proved to be a good alternative.

Numerous studies have been conducted on graft preparation techniques for ACL reconstruction.^7^^,^^16^^,^^17^ Conte et al.^18^ have suggested that grafts smaller than 8 mm in diameter present high failure rates, and according to Figueroa et al.,^19^ increasing the diameter of the graft by just 0.5 mm can lead to statistically significant improvements in the success and longevity of the graft.

The goal of the triple braid of hamstring graft is to increase the diameter of the graft, with care taken not to excessively shorten the length of the graft. This graft configuration satisfactorily reconstructs the ACL while leaving a free GT strand for the reconstruction of the ALL.

Other theoretical advantages of the technique of braiding hamstring autografts include obtaining a uniform tape-like graft, which seems to replicate the native shape of the ACL and mimic its mechanical behavior,^20^ as well as compensating for the intrinsic viscoelasticity related to soft-tissue grafts, minimizing stretching after reconstruction that eventually ends in laxity and rerupture.^21^ The main limitation of this technique is that the graft, after braiding, is shortened by approximately 5 to 10 mm.^21^ Therefore, in cases of very short grafts, this technique is not advised.

In many centers, especially those without access to a tissue bank, a major constraint of ligament reconstructions is the availability of graft material. With a braided configuration, a triple graft can have similar strength to a quadruple graft while still having a “strand” available for the reconstruction of the ALL. Technically speaking, the braid is not difficult to execute, requiring only a small learning curve.

## Disclosure

All authors (D.A.d.L., C.P.H., S.M.d.G.C.) declare that they have no known competing financial interests or personal relationships that could have appeared to influence the work reported in this paper.

## Supplementary Data

ICMJE author disclosure forms

ICMJE author disclosure forms

ICMJE author disclosure forms

Video 1 Technique for anterior cruciate ligament (ACL) and anterolateral ligament (ALL) reconstruction using triple-strand braided hamstring graft for ACL and strand for ALL reconstruction with single femoral tunnel.
