# Supplementary material for: Combined Reconstruction of the Anterior Cruciate Ligament and Anterolateral Ligament: Triple-Strand Braided Hamstring Graft for the Anterior Cruciate Ligament and Gracilis Strand for the Anterolateral Ligament With a Single Femoral Tunnel
Source: Arthrosc Tech. 2024 May 14;13(8):103023. doi: 10.1016/j.eats.2024.103023 (PMC11369946; doi:10.1016/j.eats.2024.103023)
Supplement: ICMJE author disclosure forms [file mmc1.docx]

**Declaration of interests**

**Diego Ariel de Lima**

arieldelima.diego@gmail.com

Orcid: 0000-0002-5702-1529

☒ The authors declare that they have no known competing financial interests or personal relationships that could have appeared to influence the work reported in this paper.
 
☐ The authors declare the following financial interests/personal relationships which may be considered as potential competing interests:

TITLE

Combined Reconstruction of the Anterior Cruciate Ligament and Anterolateral Ligament: Triple-Strand Braided Hamstring Graft for the ACL and Gracilis Strand for the ALL, with a Single Femoral Tunnel
